# Enhancing prefrontal modulation by phase-locking intermittent theta burst stimulation to a concurrent transcranial alternating current stimulation

**DOI:** 10.1162/imag_a_00415

**Published:** 2025-01-03

**Authors:** Nadja Zimmermann, Thomas Koenig, Andrea S. Riesen, Yosuke Morishima

**Affiliations:** Translational Research Center, University Hospital of Psychiatry and Psychotherapy, University of Bern, Bern, Switzerland; University Hospital of Child and Adolescent Psychiatry and Psychotherapy, University of Bern, Bern, Switzerland; Graduate School for Health Sciences, University of Bern, Bern, Switzerland

**Keywords:** theta burst stimulation, transcranial alternating current stimulation, TMS-EEG, prefrontal excitability, oscillations, theta rhythm

## Abstract

Theta burst stimulation (TBS) modulates cortical excitability by applying bursts of transcranial magnetic stimulation (TMS) in theta rhythms. Individual responses to TBS vary however greatly due to various factors, such as anatomical differences or the phase of the ongoing oscillatory activity in which TBS pulses are applied. To combat this variability, we exploit the ability of transcranial alternating current stimulation (tACS) to shape the state of cortical excitability in a phase-dependent manner. While cortical excitability is increased at crests of the tACS-induced current, applying the TBS triplet pulses at these crests has the potential to produce larger neuronal responses and thus increase the likelihood of long-term potentiation (LTP). In our randomized sham-controlled study, we focused on enhancing prefrontal cortex excitability by phase-locking intermittent TBS (iTBS) to the crests of an induced 5 Hz tACS current. Twenty-seven healthy participants received two iTBS sessions, once paired with sham-tACS and once with active tACS in a cross-over design. We evaluated effects of our stimulation protocol on cortical excitability by comparing TMS-induced activity and resting-state Microstates in the EEG before and after the stimulation as well as between the two sessions. We found significant effects of iTBS on channel-wise, global, and oscillatory TMS-induced activity, as well as changes in Microstates. The concurrent, phase-locked tACS-iTBS protocol notably decreased the N100 amplitude of the Global Mean Field Power. We also found that baseline TMS-induced oscillatory activity was a key predictor of changes in TMS-related oscillatory activity. In the case of TMS-related gamma oscillations, a significant interaction between our stimulation protocols and baseline activity was observed, indicating that the relationship between baseline and post-iTBS oscillations was strengthened by the concurrent phase-locked tACS-iTBS stimulation protocol. These findings highlight the potential of phase-locked tACS to enhance the effects of iTBS on prefrontal cortical excitability.

## 1 Introduction

Repetitive transcranial magnetic stimulation (rTMS) is a non-invasive brain stimulation method to induce sustained changes in cortical excitability outlasting the stimulation duration. Depending on the sequence of rTMS used, inhibitory effects suggested to mimic long-term depression (LTD) or excitatory effects suggested to mimic long-term potentiation (LTP) of synaptic strength can be induced (Klomjai et al., 2015). Theta burst stimulation as a special form of rTMS applies stimulation in a manner mimicking endogenous theta rhythms in the hippocampus (Huang et al., 2005). Compared to traditional rTMS protocols which take 20–40 minutes of application time, TBS protocols only take around 3–4 minutes. Although application time is drastically reduced, there is a high Inter-individual variability whether iTBS produces facilitatory changes in brain excitability or not. Some of the possible reasons which have been discussed as being the source of this problems are age (Guerra et al., 2020), anatomy (Hamada et al., 2013), baseline response to TMS (Corp et al., 2020), or the phase of ongoing oscillations in the stimulated brain region (Baur et al., 2020).

Some studies attempted to solve this last problem by employing a closed-loop system, triggering TMS pulses according to the ongoing neural oscillations using EEG (Ding et al., 2022;Faller et al., 2022). While these protocols seem promising, they require a more advanced algorithm and setup for their application. Other studies sought to boost LTP-like plasticity in a simpler manner by concurrently applying transcranial alternating current stimulation (tACS) and TBS (Guerra et al., 2018;Maiella et al., 2022). TACS is the application of weak alternating currents to the scalp, which can entrain ongoing brain oscillations to synchronize with the induced activity. This increases oscillatory power in the corresponding frequency band (Helfrich et al., 2014;Kanai et al., 2008;Zaehle et al., 2010). It has been hypothesized that tACS works by increasing or decreasing membrane potentials depending on the phase of the current, making de- or hyperpolarization more likely to happen (Vöröslakos et al., 2018).Guerra et al. (2018)concurrently applied either gamma or beta tACS and iTBS to the primary motor cortex (M1). They observed boosted and prolonged effects of iTBS on cortical excitability after pairing iTBS with gamma tACS, but not with beta tACS.Maiella et al. (2022)evaluated the effects of pairing iTBS to the dorsolateral prefrontal cortex (DLPFC) with tACS at gamma or theta frequency and found an increase in gamma power and local connectivity after applying iTBS and gamma tACS.

An important property of tACS not considered in these studies is its ability to shape cortical excitability in a phase-dependent manner. Application of TMS at crests of an induced theta tACS current resulted in a heightened excitability of the DLPFC compared to the application at troughs (Fehér et al., 2017). Following these results, tACS applied at theta frequency with an intensity of 1 mA could be utilized as a priming instrument for TBS. While cortical excitability is increased at crests of the tACS induced current, applying TBS in sync with the crests has the potential to improve the effectiveness of TBS protocols. Evaluating the effect on cortical excitability of such a concurrent phase-locked tACS-iTBS protocol will be the main goal of the present study. The prefrontal cortex is well suited for the application of this protocol based on its endogenous theta rhythm, which can be entrained by theta frequency tACS. Neurophysiological studies have elucidated that property of neural networks are distinct among brain areas, with TMS applied to different areas evoking alpha-band oscillations in the occipital cortex, beta-band oscillations in the parietal cortex, gamma- as well as beta-band oscillations in the frontal cortex (Rosanova et al., 2009), and theta-band in the prefrontal cortex (Nigbur et al., 2012). Additionally, the DLPFC plays an essential role in a wide range of functions, including implementing executive control (Gbadeyan et al., 2016;MacDonald et al., 2000;Ridderinkhof et al., 2004) and the regulation of emotions (Drabant et al., 2009;Goldin et al., 2008). Dysfunction of the prefrontal cortex has been observed in psychiatric populations, which is why TBS on the left DLPFC has been widely applied to treat patients (Lefaucheur et al., 2020).

A recently published study byBriley et al. (2024)employed the same phase-locked iTBS-tACS stimulation protocol as used here. They assessed the effects on brain plasticity by evaluating EEG resting-state and n-back task data. The stimulation protocol resulted in an enhancement of frontal theta power measured during the n-back task increasing over the 15-minute post-stimulation period. Not assessed in that study was the effect of this stimulation protocol on TMS-EEG measures. Cortical excitability can be probed directly by applying single-pulse TMS to the area of interest and measuring the evoked activity with EEG (Ilmoniemi & Kičić, 2010). This has been utilized previously in a variety of studies to quantify the effects non-invasive brain stimulation methods have on neural excitability, although results vary.Cruciani et al. (2023)summarized effects of various neuromodulation techniques on TMS-EEG outcomes in a review. Regarding TMS-evoked potentials (TEPs), iTBS to the DLPFC increased both N100 (Chung et al., 2017,2018a,2018b) and P200 (Chung et al., 2017,2018a) component amplitudes and increased theta band power in healthy subjects (Chung et al., 2017,2018b). Other studies produced opposite findings, with iTBS resulting in decreased N100 (Chung et al., 2019) and P200 (Chung et al., 2019;Desforges et al., 2022;Luo et al., 2023) amplitudes and a decrease in theta power (Desforges et al., 2022).

The aim of the present study was to implement a modified iTBS stimulation protocol to boost LTP effects by applying the iTBS in a phase-locked manner at crests of an induced tACS current. The effects of this stimulation protocol on prefrontal excitability were evaluated by comparing TMS-evoked activity in the EEG and resting-state EEG before and after administration of the protocol and compared to a sham stimulation.

Changes in brain excitability could potentially manifest in different aspects of activity measured with EEG. While we stimulate locally, we can assume that any induced changes will also affect the network level. In the case of our TMS-EEG data, we therefore analyzed TEPs, Global Mean Field Power (GMFP), and TMS-related oscillations. While the GMFP captures more global changes in brain excitability by quantifying the standard deviation across all electrodes, channel-wise analysis on TEPs provides insight into more localized changes. TMS-related oscillatory activity, on the other hand, represents the frequency aspect of EEG data. Additionally, effects on resting-state EEG were assessed by conducting a microstate (MS) analysis. MS refer to topographical maps of electric potentials which remain stable for 80–120 ms (Lehmann et al., 1987). MS can capture states of global networks, representing brain dynamics which are often affected in neuropsychiatric diseases (Michel & Koenig, 2018). With MS analysis we can therefore assess systemic changes in the resting-state EEG. Other studies have thus far found changes after administration of different stimulation protocols such as rTMS or continuous TBS (cTBS) in patients (Ding et al., 2023;Gold et al., 2022;Guo et al., 2023;Pan et al., 2021;Sverak et al., 2018;Zhao et al., 2023) but also in healthy participants (Croce et al., 2018;Qiu et al., 2022).

## 2 Methods

### 2.1 Participants

Twenty-seven right-handed participants (16 female, 11 male) aged between 18 and 29 (m = 24.16, SD = 3.49) were included in the study. All participants were screened for contraindications to TMS with the TMS-safety screening (Rossi et al., 2009,^2011^) and excluded in case of a present or past diagnosis of a psychiatric or neurological disorder as well as substance abuse in the 4 weeks before the study and intake of psychoactive medication. Each participant underwent two sessions of iTBS, once coupled with phase-locked tACS and once with sham tACS. The safety screening was assessed again at the start of both sessions to account for any changes in the time between. Participants received 50.- CHF per study session, amounting to 100.- CHF in total for the two sessions as compensation. They were pseudorandomized into two groups deciding which condition they will receive first. The second session took place at least 2 weeks later to avoid any carry-over effects.

The study was approved by the Kantonale Ethikkommission (KEK) Bern (2021-D0087) and conducted in compliance with the Declaration of Helsinki. Written informed consent from each participant was obtained before the start of the first session.

### 2.2 Procedure

#### 2.2.1 EEG acquisition

Active TMS motor threshold (aMT) was determined with a TMS stimulator (MagPro R30, Tonica Elektronik A/S, Lucernemarken, Denmark) and a figure of 8 coil (C-B60, Tonica Elektronik A/S, Lucernemarken, Denmark). Subjects were sitting on a chair with their hand on an armrest, index finger slightly elevated, and EEG cap (BrainCap TMS, Brainproducts, Gilching, Germany) already mounted. TMS output intensity eliciting a finger twitch five out of 10 times was determined as aMT.

After assessing the aMT, electroconductive EEG gel was applied to reduce the impedance between electrodes and the scalp. Impedances were checked throughout the measurements and kept sufficiently low (around 5 kΩ). A 64-channel EEG system (Brainamp DC, Brainproducts, Gilching, Germany) was used to measure the EEG, with electrode FCz as the online reference and AFz as the ground electrode and a sampling rate of 2,500 Hz. To avoid saturation of the amplifier due to a drift in baseline, a direct current (DC) correction was applied automatically when saturations reached 75%.

As a first measurement, resting-state EEG data were acquired for 120 seconds while participants sat in a chair with their eyes open, fixating a cross on the wall to reduce eye-movement artifacts.

**Fig. 1.**
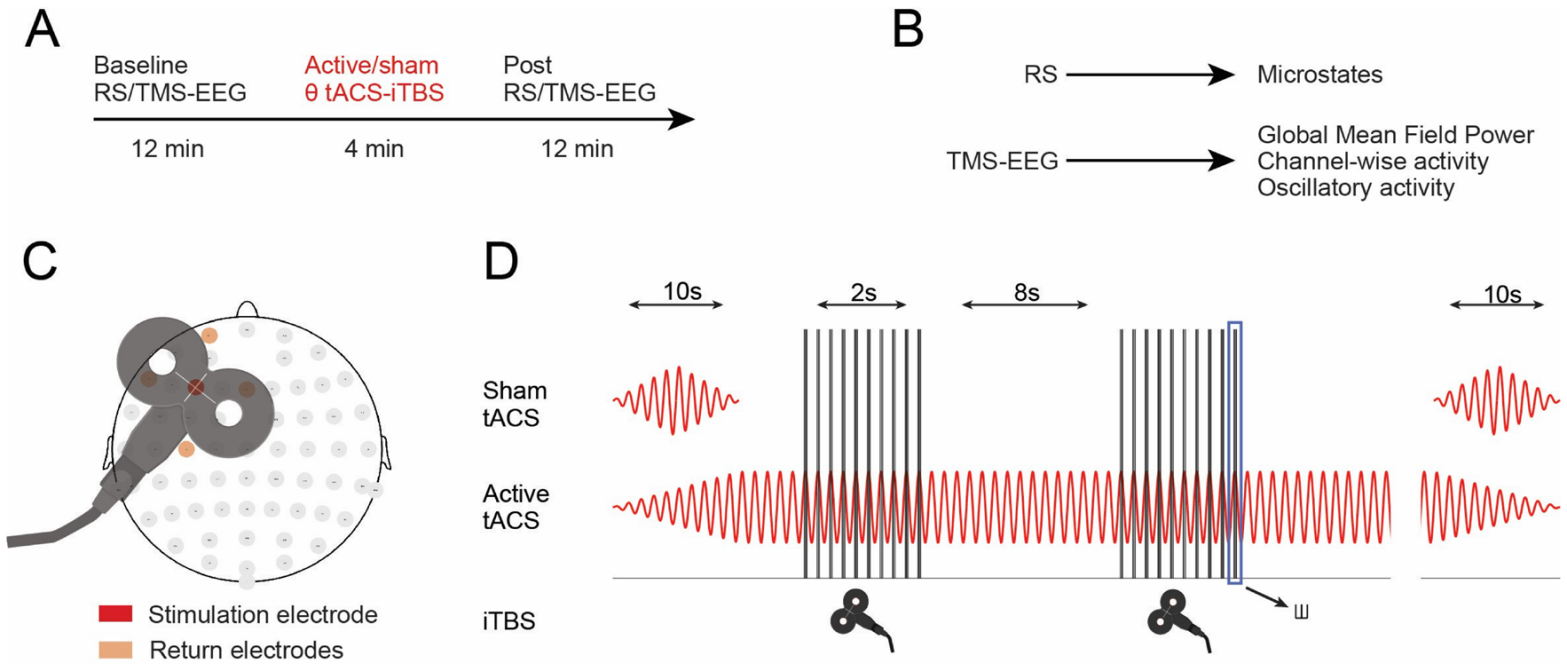
Experimental procedure, electrode montage, and stimulation protocols. (A) Procedure of the experimental session. As a baseline measurement, resting-state (RS) EEG was measured for 2 minutes, followed by sub- and supra-threshold single-pulse TMS-EEG each taking around 5 minutes. Next was either sham- or active tACS-iTBS stimulation for 4 minutes. The same RS and TMS-EEG measurements were repeated post-stimulation. (B) Analyses conducted on the RS and TMS-EEG measurements. (C) Depiction of the targeted cortical area. Electrode F3 was picked as the target location for single-pulse TMS and iTBS as well as the stimulation electrode for tACS. Electrodes Fp1, Fz, F7, and C3 acted as return electrodes for the tACS. (D) During sham tACS-iTBS, the tACS current is ramped up and down again over 10 seconds in the beginning and iTBS is applied without a concurrent tACS stimulation. In the active condition, the tACS current is ramped up and kept consistent until the end of the stimulation. The iTBS is applied in phase at the crests of the tACS current.

After the resting-state, single-pulse TMS-EEG was measured at both supra- (110% of aMT) and sub-threshold intensity (90% of aMT). For both intensities, single-pulse TMS were applied 100 times with 3 seconds of inter-stimulus-interval (with jittering) to the left DLPFC. The TMS coil was fixated in a tripod and placed over electrode F3, while the handle was pointing 45° away from the midline to the left (Fig. 1C). All three measurements (resting-state, sub- and supra-threshold TMS-EEG) were obtained again after the tACS-iTBS stimulation.Figure 1Aprovides an overview of the experimental sessions, whileFigure 1Bdepicts an overview of the analyses conducted on the respective EEG data type.

To reduce auditory evoked potentials associated with the TMS coil clicking sound and protect their hearing, subjects listened to white noise through noise-canceling in-ear earbuds during TMS-EEG measurements and the tACS-iTBS stimulation. While this decreased the audibility of the clicking sound, participants reported that they were still able to hear it.

#### 2.2.2 Phase-locked tACS-iTBS

To apply tACS, high-definition-tACS (HD-tACS,Villamar et al., 2013) montage and a DC-Stimulator Plus (NeuroConn GmbH, Germany) was used. The frequency of tACS was set at 5 Hz (theta frequency) with a peak-to-peak intensity of 1 mA to increase excitability at the peaks (Fehér et al., 2017). The tACS current was applied through the EEG electrodes, which were sintered Ag/AgCl electrodes with an inner opening of approximately 6 mm. To this end, the electrodes at locations F3, AF3, F1, FC3, and F5 were disconnected from the EEG and reconnected to the DC-Stimulator, with the F3 electrode serving as the stimulation electrode and the rest as the return electrodes as seen inFigure 1C. After the tACS-iTBS protocol, the electrodes were reconnected to the EEG. The iTBS was applied over electrode F3 with the same TMS stimulator and coil used during TMS-EEG measurement, and TMS intensity was set to 90% of aMT. Both TMS and tACS were controlled by National Instruments analogue board (M/N PCI-6722) and Matlab with data acquisition toolbox (https://ch.mathworks.com/de/products/data-acquisition.html) to ensure the precise timing of synchronized stimulation. The details of the active and sham tACS-iTBS protocols are depicted inFigure 1D. In the active condition, the 5 Hz tACS current was ramped up to its full peak-to-peak amplitude over the span of 10 seconds, kept at this intensity for 220 seconds, and subsequently ramped down to zero for 10 seconds. The iTBS was programmed to start 30 seconds after starting the tACS current. A triplet of 50 Hz TMS pulses was delivered every 200 ms (milliseconds), and the second pulse of the triplet was aligned to the crest of the 5 Hz tACS waveform. After 2 seconds containing 10 sets/30 TMS pulses, there was a break of 8 seconds. This was repeated 20 times, amounting to 600 TMS pulses in total within a duration of about 4 minutes, including the initial tACS period.

Application of tACS could produce a tingling and itching sensation at stimulation sites at the beginning of stimulation. Therefore, in the sham tACS-iTBS condition, we introduced the identical 5 Hz tACS current with 1 mA peak-to-peak intensity that was ramped up and immediately down again at the beginning of the sham stimulation over a span of 10 seconds, followed by a 20-second break before starting the iTBS protocol. Applying the tACS current for such a short amount of time has been found to not result in significant after-effects on cortical excitability (Dissanayaka et al., 2018). At the end of each session, participants were asked about their subjective feeling whether they think they received active-tACS or sham-tACS. In the first session, 16 out of 27 participants judged correctly which stimulation condition they received. In the second session, 20 out of 27 were correct. To test whether this was significantly different from a chance of 50% of participants guessing the answer, a binomial test was conducted. The observed proportion of participants guessing correctly was non-significant in the first session (*p*= 0.442), but significant in the second session (*p*= 0.019). This suggests that participants were able to correctly discriminate the sham and active conditions in the second session.

#### 2.2.3 EEG preprocessing and analysis

EEG data were preprocessed offline in Matlab (R2021a, MathWorks, Inc., Natick, MA, United States) using EEGLAB (v14.1.2.,Delorme & Makeig, 2004;https://sccn.ucsd.edu/eeglab/index.php) and tmseeg (v5.0,Atluri et al., 2016;https://github.com/EEGSignalProcessing/TMSEEG/releases/tag/v5.0) toolboxes and subsequently analyzed with custom scripts, microstatelab (Nagabhushan Kalburgi et al., 2023;https://sccn.ucsd.edu/eeglab/plugin_uploader/plugin_list_all.php) plugin and RStudio (https://posit.co/downloads/).

TMS-EEG data were segmented into epochs from -500 to 500 ms in relation to the TMS pulse, and baseline was set to -200 to -2 ms. Mastoid channels (TP9 and TP10) were removed due to excessive muscle artifacts. Data were manually inspected and epochs with excessive noise and eye blinks in the time period -200 to 400 ms relative to the TMS pulse removed. An average number of 75 epochs were kept from the 100 single-pulse TMS epochs for further analyses. If a channel showed electrode artifacts or if excessive decay of the TMS artefact was localized to one channel, it was interpolated. The reference was set to the average reference. Then to remove the initial TMS artifact and its associated decay, the TMS pulse was cut with the tmseeg toolbox from -1 to 7 ms around the TMS pulse and leftover decay was removed by ICA. The missing 8 ms of data was later linearly interpolated. Some participants produced very small blink artifacts right after the TMS pulse, in addition to regular blinks at later timepoints. When the larger regular blinks—which usually occurred at later time points after 500 ms—coexist with the smaller blinks within epoched data, the immediate small TMS-induced blinks were not effectively distinguished from other brain activities by ICA. As a result, we chose to cut the epochs from -500 to 500 ms relative to TMS onset to exclude the late larger blinks, which improved the removal of the smaller blinks by ICA. The mean number of ICA components removed overall was 0.98. Two participants were fully excluded for data analysis due to blinking artifacts not being fully removed after preprocessing. Another participant was excluded from TMS-EEG data analysis due to unexpectedly large TMS-evoked response, where 23 out of a total 80 time bins were detected as an outlier by the MATLAB function “isoutlier” during GMFP analysis. This function classifies a value as an outlier if it is more than three scaled median absolute deviations from the median. This resulted in*N*= 24 for TMS-EEG analyses and*N*= 25 for resting-state analysis.

##### 2.2.3.1 TMS-evoked potentials

To obtain TMS-evoked potentials (TEPs), data were first lowpass filtered at 100 Hz and subsequently averaged across trials for each participant separately for each recording condition. During this step, we noticed that baseline correction had to be adjusted to -40 to -2 ms. The GMFP (Lehmann & Skrandies, 1980) was calculated as the standard deviation across all channels to assess the overall magnitude of the TEPs across the whole brain as follows.

$$\text{GMFP}\left(t\right)=\sqrt{\frac{\sum_{i}^{k}{\left(V_{i}\left(t\right)-V_{mean}\left(t\right)\right)}^{2}}{k}}$$

This was done for each subject and recording condition separately using the TEPs. The resulting datasets were then subsequently binned by calculating the mean over time periods of 20 ms, starting 20 ms before TMS onset until 400 ms after the TMS pulse. Repeated measures (RM) two-way ANOVAs were calculated for each time bin starting from 40 ms onwards of the GMFP with the factors of time (pre/post stimulation) and condition (active/sham session). For channel-wise analysis, the TEPs were again downsampled and RM two-way ANOVAs were calculated for each electrode and time bin with the factors time (pre/post stimulation) and condition (sham/active session). To protect against false positives due to multiple testing across electrodes and time points, a TANOVA with 1,000 permutations was computed with the MATLAB toolbox Ragu (Randomization Graphical User interface,Habermann et al., 2018,https://www.thomaskoenig.ch/index.php/work/ragu/1-ragu) for each sample point with the factors condition (active/sham) and time (pre/post), followed up by an overall test of the duration of time-periods with*p*-values below 5% (Koenig & Melie-Garcia, 2010). Only time periods where this duration exceeded a 5% chance level were considered further. To limit the analysis to topographic effects independent of GMFP differences, all data were normalized to GMFP = 1 before the computation of these TANOVAs.

##### 2.2.3.2 TMS-related oscillations

To extract total oscillatory activity elicited by TMS, the 1,000 ms of TEPs were first bandpass filtered from 3 to 100 Hz and subsequently Morlet wavelet decomposition was performed. The cycles were set to logarithmically increase from 3 to 10 and frequencies extracted started from 3 to 70 Hz logarithmically increasing in 30 steps. Afterward, the resulting datasets were downsampled to steps of 20 ms and averaged over all trials to obtain the total oscillatory response to TMS. Conversion to decibel (dB) to normalize the data was achieved by dividing all the data by a mean baseline value from -400 to -200 ms and multiplicate by 10 times its logarithm. This was done for each electrode, frequency and dataset separately.

To compare time-frequency data between conditions, the mean over all participants from both active and sham baseline conditions of the supra-threshold data was plotted to search for electrodes of interest (Fig. 5A). Then, we defined time and frequency ranges (Fig. 5B) based on the peak of power values. For theta, this resulted in a time-window from 100 to 240 ms and 4 to 7 Hz and electrodes C1, Cz, C2, FC1, FCz, and FC2. For beta the time and frequency window was 0 to 60 ms and 15 to 21 Hz and for gamma -20 (before stimulus onset due to temporal smudging) to 40 ms and 30 to 70 Hz. For both beta and gamma, the electrodes of interest were AF3, F3 and F1. For all datasets and each frequency band separately, power was extracted and averaged over the respective time-, frequency- and electrode-range of interest. An RM three-way ANOVA was conducted in R with the factors time (pre/post), condition (active/sham) and intensity (sub/supra) to assess general main- and interaction effects. In the case of a significant interaction effect, Wilcoxon signed rank tests were computed as post-hoc tests (due to a non-Gaussian distribution of power data) to gain an insight into the direction of the obtained effects. This was done separately for frequency band, condition, and intensity.

Additionally, visual inspection of power at baseline and their changes after the stimulation protocols from each participant revealed large interindividual differences. As shown in previous studies, baseline activity could predict subsequent iTBS effects on the primary motor cortex using motor-evoked potentials (Corp et al., 2020;Leodori et al., 2021). Similarly, to evaluate whether baseline (pre-iTBS) power values in the corresponding frequency band could significantly predict changes in post-iTBS, robust linear regressions with Huber weights were performed as post-hoc tests. Changes were evaluated as a change score subtracting baseline power values from post-iTBS power values per subject. The model included baseline power, condition (active/sham), and interaction terms between the predictor variables. The predictor variables were mean centered. This was analyzed for each frequency band (theta, beta and gamma) and each measurement (TMS intensity at sub- and supra-threshold) separately.

##### 2.2.3.3 Microstate analysis of resting-state EEG

In addition to TMS-EEG data, we evaluated the effects of the tACS-iTBS protocol on brain activity by using resting-state EEG data. To this end, we applied MS analysis to compare between before and after active and sham tACS-iTBS.

Analog to the TMS-EEG data, mastoid channels (TP9 and TP10) were removed and time periods with DC correction artifacts were manually cut from the resting-state EEG data. Afterward, data were bandpass filtered from 2 to 30 Hz and the reference set to average. ICA decomposition was achieved by using the EEGLAB runica function. Components representing eye blinks and eye movements were subsequently removed (mean removed = 2.18). To obtain MS information, the GMFP of the resting-state data was first calculated for each subject and condition. Topographic maps were extracted from the GMFP peaks and subsequently clustered using k-means in each subject and condition.

**Fig. 2.**
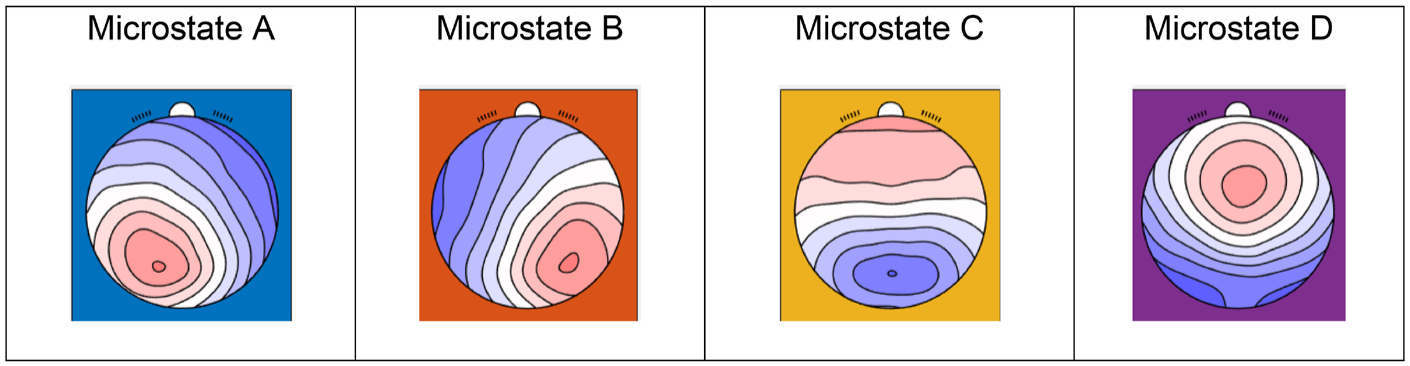
Topographic maps of the obtained canonical Microstates (A, B, C and D).

Grand mean maps were calculated for each class by averaging maps in the clusters across all subjects and all conditions. In the current analysis, we chose the four-class solution of the MS, because of following reasons. First, the mean shared variance of the grand-mean template maps with the individual template maps was highest (94.8%) in the four-class solution. Second, MS maps of our four-class solution were very similar to typical MS maps and it is therefore easier to relate our finding to previous studies. The maps were sorted automatically with a template (Custo et al., 2017) to enable comparison, labeling the maps as MS A, B, C, and D (Fig. 2). These grand mean maps were then backfitted onto the individual datasets and descriptive parameters of Occurrence, Duration, and Coverage were extracted for each MS. Occurrence represents the average number of times an MS occured per second, duration the average time in milliseconds before an MS switched to the next, and coverage how much percent of the total recording time an MS was active.

An RM two-way ANOVA with the factors time (pre/post) and condition (active/sham) was performed in MATLAB separately for all descriptive parameters (Occurrence, Duration, and Coverage) and MS (A, B, C, and D) to assess main- and interaction effects.

## 3 Results

### 3.1 Gmfp

We first evaluated changes of TMS-evoked activity across all channels induced by active and sham tACS-iTBS. Evaluating the effects of time (baseline vs. post) and condition (active vs. sham) by RM two-way ANOVA on the global magnitude of the neuronal response (GMFP) after sub-threshold stimulation resulted in a significant interaction effect between 120–140 ms (*F*(1,23) = 3.686,*p*= 0.009) as well as significant main effects of time between 200–260 ms (*F*(1,23) = 4.669,*p*= 0.041;*F*(1,23) = 5.463,*p *= 0.028;*F*(1,23) = 4.803,*p*= 0.039), and between 320–380 ms (*F*(1,23) = 10.219,*p*= 0.004;*F*(1,23) = 16.021,*p*<0.001;*F*(1,23) = 10.105,*p*= 0.004), depicted inFigure 3. In the case of the supra-threshold stimulation, the RM two-way ANOVA resulted in a significant main effect of time between 320–380 ms (*F*(1,23) = 5.120,*p*= 0.033;*F*(1,23) = 6.771,*p*= 0.016;*F*(1,23) = 5.954,*p*= 0.023) but no interaction effect (seeSupplementary Fig. 1).

**Fig. 3.**
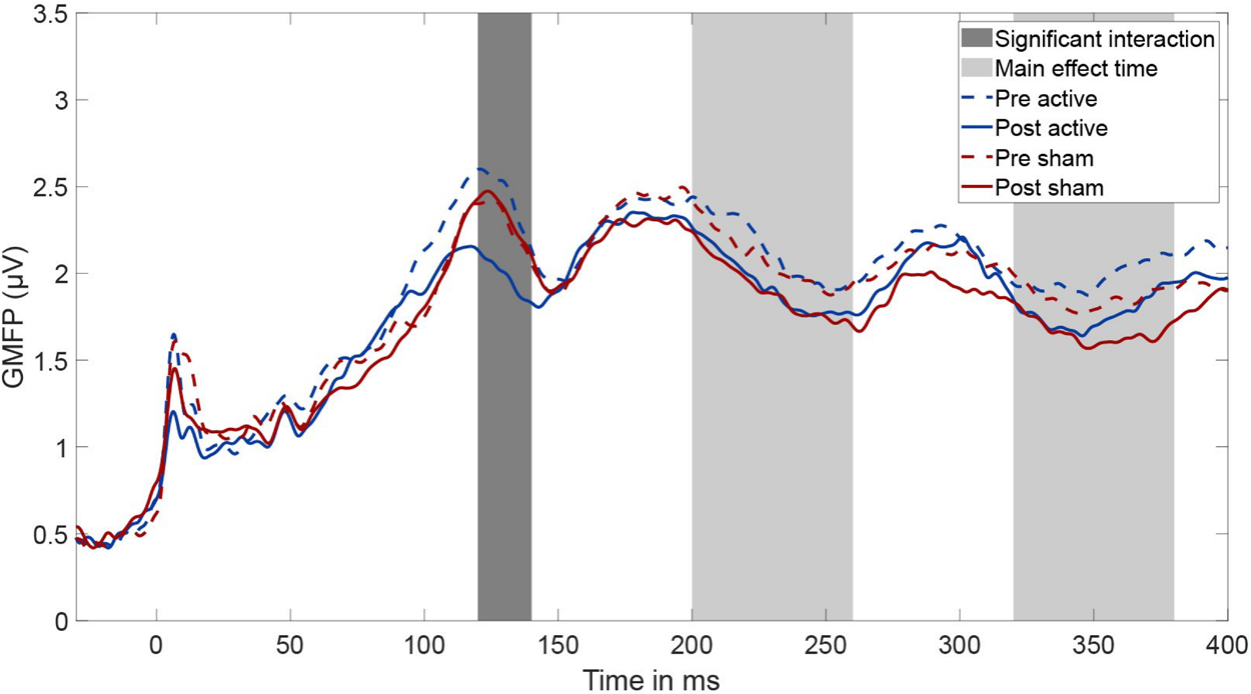
Global Mean Field Power after sub-threshold TMS. Global Mean Field Power of TMS-evoked potentials across the scalp. Baseline (pre-) and post-iTBS are depicted separately for the active and sham conditions. Time periods marked in light grey depict a significant main effect of the active and sham stimulation conditions, whereas the dark grey area depicts a significant interaction effect between time and condition.

### 3.2 Electrode-wise analysis of TEPs

The result of the channel-wise, RM two-way ANOVA to assess localized effects of the active and sham stimulation protocols are presented inFigure 4. After an initial positivity of potentials at the site of stimulation, this spreads to a more fronto-central location where potentials turn negative. This pattern is in the typical time- and channel- region of an N100 component. Potentials subsequently turned positive in a central area, representing a P200. The supra-threshold stimulation resulted in a very similar pattern with a slightly increased intensity (Supplementary Fig. 2). The TANOVA analysis was conducted on sub-threshold data, yielding several time intervals with*p*< 0.05 for the factor time, but only a late (84.8–200 ms) time window was longer than what was expected at a 5% chance level (which was at 24.4 ms). There were no time points with*p*< 0.05 for condition, and a very brief (2 ms) interval with*p*< 0.05 for the interaction, which was far shorter than the duration expected at a 5% chance level (which was at 17.2 ms). We, therefore, retained the null hypothesis of no significant topographic interaction of time and condition.

**Fig. 4.**
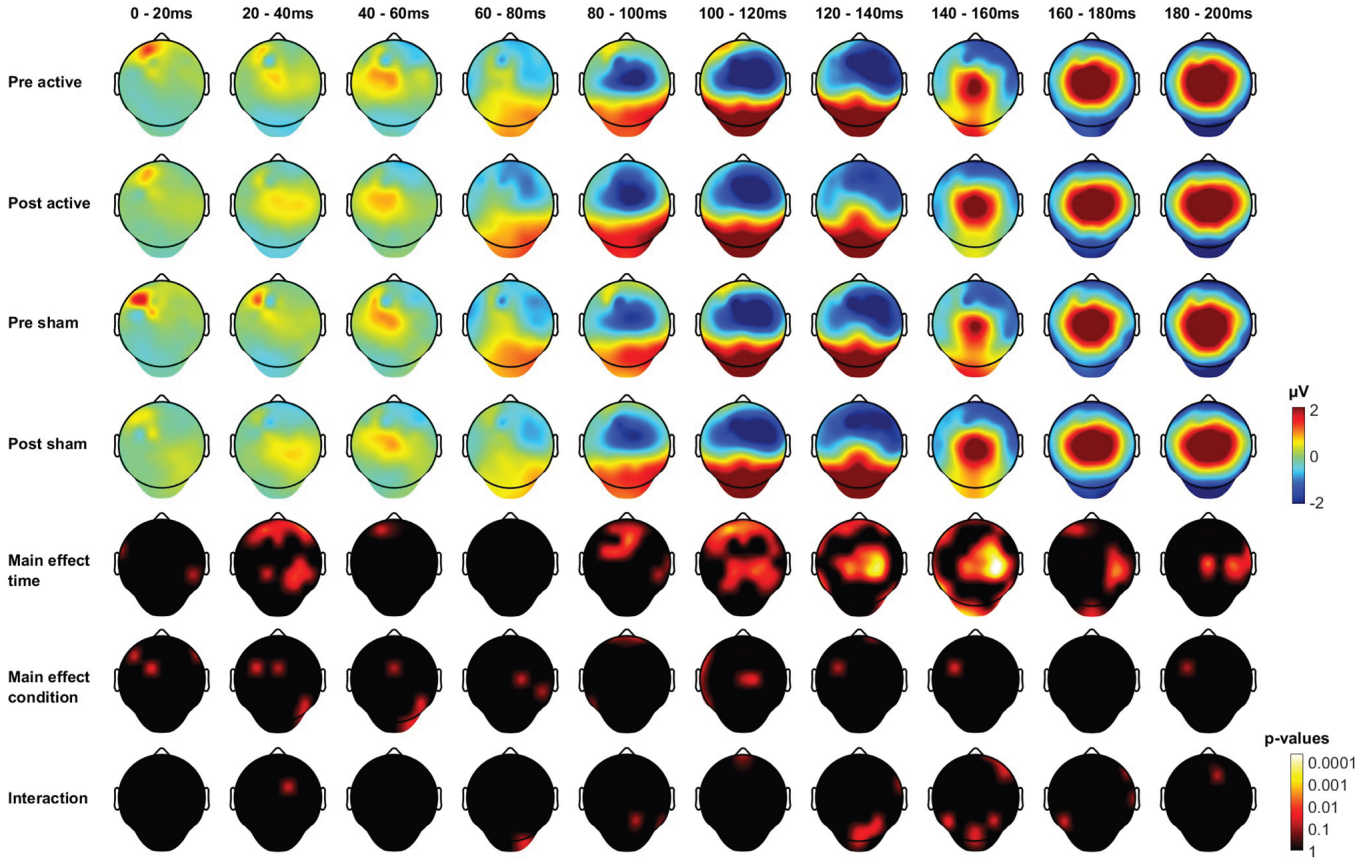
Results of electrode-wise analysis. Depiction of sub-threshold TMS-evoked potentials across the scalp per measurement condition and results of the electrode-wise RM two-way ANOVA.

Consistent with the TANOVA statistics, electrode-wise post-hoc RM two-way ANOVA suggest no relevant main effects of condition and no interaction, since only some individual electrodes turned significant for a period of 20 ms. In regard to a main effect of time some more evident patterns emerged. The largest differences emerged in a time range between 100–160 ms after the TMS, where potentials seem to be more positive in the post-iTBS measure. Since this effect is in the time range of the N100, this represents less negative TEPs around the vertex. In the case of the supra-threshold data, the main effect of time was visibly reduced compared to the sub-threshold intensity. Similarly, no clear interaction effect was found (Supplementary Fig. 2).

### 3.3 Oscillations

**Fig. 5.**
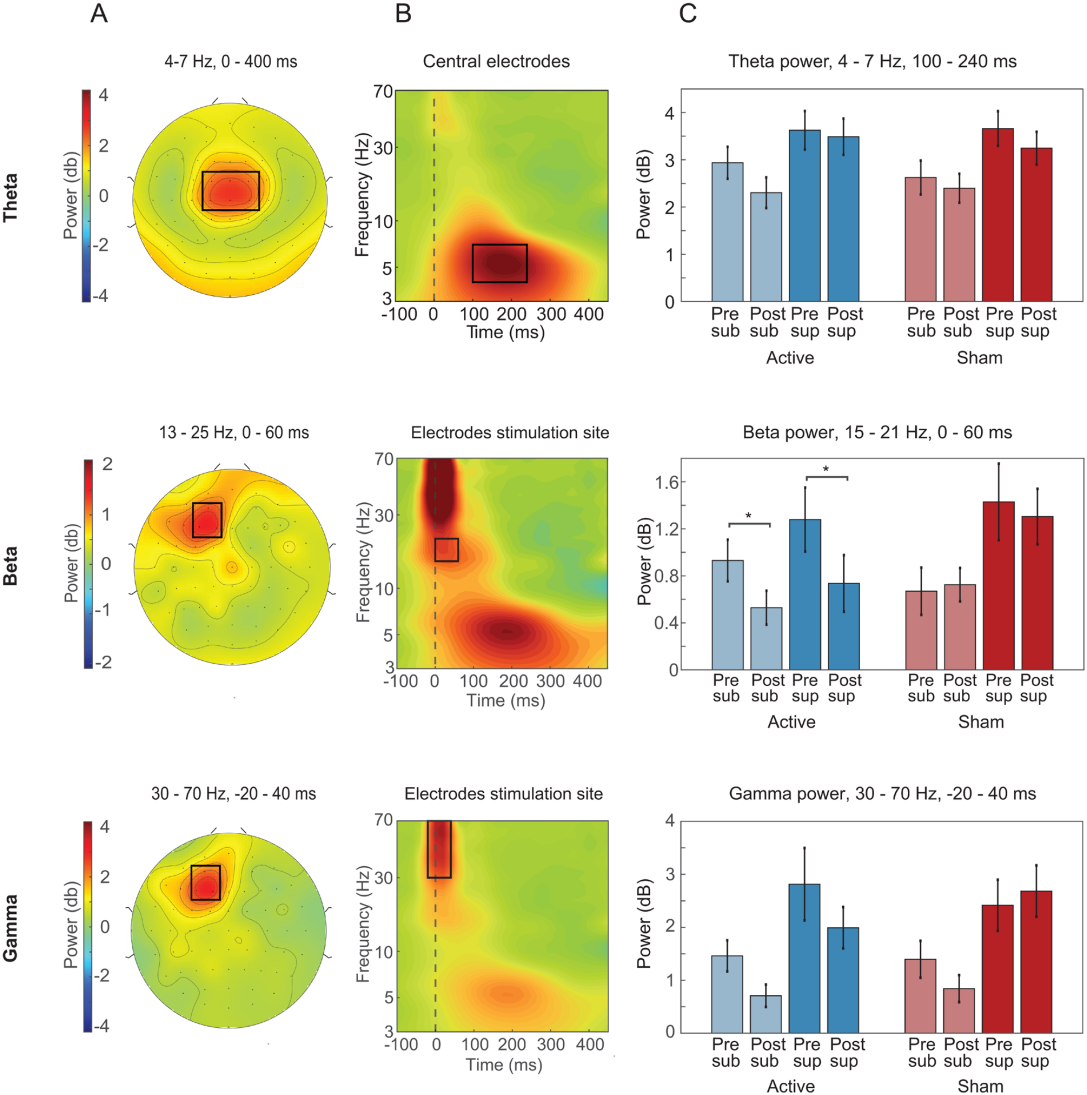
TMS-related oscillations. (A) Topographical plots depicting distribution of oscillatory power at baseline from both active and sham in the supra-threshold condition to choose electrodes of interest: AF3, F1, and F3 for beta and gamma power and C1, Cz, C2, FC1, FCz, and FC2 for theta power. (B) Averaged oscillatory power across electrodes of interest to determine time frame of interest. (C) Oscillatory power per frequency band extracted from the electrodes and time of interest. Error bars depict standard error of the mean. Significant differences obtained from Wilcoxon signed rank tests are depicted for the beta frequency band. *p≤ 0.05.

We then evaluated TMS-related oscillatory power before and after active or sham tACS-iTBS. Changes in TMS-related oscillatory power differed from each other depending on the frequency band analyzed (Fig. 5). For theta power, the three-way ANOVA resulted in a significant effect of time (*F*(1,23) = 36.138,*p*< 0.001) and intensity (*F*(1,23) = 5.671,*p*= 0.026), with effect sizes (generalized eta squared,*η^2^_g_*) being medium for time*(η^2^_g_*= 0.07) and small for intensity (*η^2^_g_*= 0.01). For gamma power, results were similar with a significant effect of time (*F*(1,23) = 25.761,*p*<0.001) and intensity (*F*(1,23) = 5.191,*p*= 0.03), with effect sizes being medium for time (*η^2^_g_*= 0.1) and small for intensity (*η^2^_g_*= 0.01). For beta power, time had a significant effect on power as well (*F*(1,23) = 15.872,*p*< 0.001) with a medium effect size (*η^2^_g_*= 0.06). The interaction between time and condition was significant as well (*F*(1,23) = 4.698,*p*= 0.041) with a small effect size (*η^2^_g_*= 0.01).

Due to the significant interaction effect in beta oscillatory power, Wilcoxon signed rank tests were utilized to gain further insights into the observed effects. In the active condition, a significant decrease in beta power was observable for both the sub-threshold (*z*= -2.678,*p*= 0.007) and supra-threshold stimulation (*z*= -2.1929,*p*= 0.028). In the sham condition, a slight nonsignificant increase in beta power emerged post-iTBS in the sub-threshold stimulation. Comparing the differences of post-pre between the active and sham condition only led to a trend for an effect (*z*= -1.897,*p*= 0.058) in the sub-threshold stimulation.

#### 3.3.1 Baseline (pre-iTBS activity) predicts iTBS-induced changes in oscillatory power

Based on the ANOVA analysis, we found a reduction of TMS-related oscillatory activity in all frequency bands after iTBS. However, there were extensive inter-individual differences in oscillatory power changes. To address the source of this heterogeneity, we calculated robust linear regression to evaluate whether baseline oscillatory power can predict changes in oscillatory power after iTBS. Previous research (Corp et al., 2020;Leodori et al., 2021) found that baseline MEP were able to predict changes after iTBS.

Calculation of the robust linear regressions revealed that in all evaluated frequency bands, baseline TMS-related oscillatory power can significantly predict power changes post-iTBS (Table 1). The interaction between baseline and condition was a significant predictor of power changes post-iTBS for the gamma frequency band in both sub- and supra-threshold measurements (*p*< 0.05). This indicates that the tACS-iTBS stimulation reinforced the relationship between baseline and change scores in gamma band activity.

**Table 1.** Results of robust linear regression.

	Intercept	Baseline	Condition	Baseline x Condition	Model *F* (df)
Theta Sub	-0.452 (0.153)**	-0.349 (0.090)***	0.455 (0.307)	0.199 (0.181)	6.5 (44)***
Theta Supra	0.824 (0.471)	-0.244 (0.114)*	-0.549 (0.698)	0.063 (0.171)	2.61 (44)
Beta Sub	-0.168 (0.081)*	-0.693 (0.099)***	0.242 (0.162)	-0.203 (0.197)	19.9 (44)***
Beta Supra	-0.522 (0.121)***	-0.689 (0.085)***	0.586 (0.241)*	0.217 (0.170)	23.4 (44)***
Gamma Sub	-0.661 (0.083)***	-0.573 (0.060)***	0.326 (0.167)	0.387 (0.120)**	33 (44)***
Gamma Supra	-0.248 (0.198)	-0.382 (0.073)***	1.151 (0.397)**	0.348 (0.145)*	19.3 (44)***

*Note.*The estimate (beta) is depicted for the intercept, the two predictors (baseline and condition), and their interaction with the standard error in brackets for each frequency band and TMS intensity separately. The last column denotes the*F*-values of the model and the degrees of freedom in brackets. *p≤ 0.05, **p≤ 0.01, ***p≤ 0.001.

Generally, lower baseline values resulted in higher values in oscillatory power post-iTBS, while higher baseline values resulted in a suppression of oscillatory power post-iTBS. This relationship is depicted inFigure 6.

**Fig. 6.**
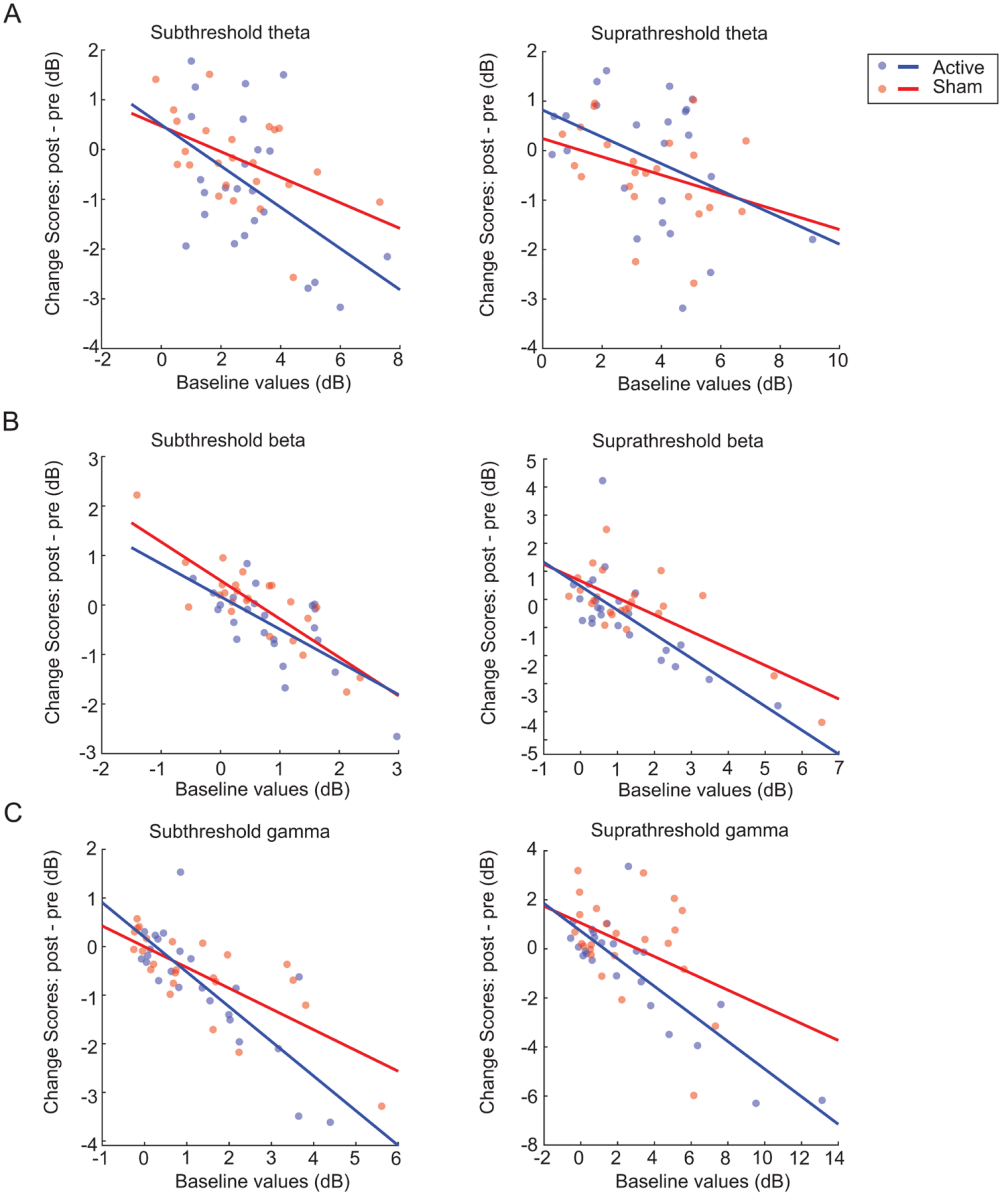
Relationship between TMS-related power values at baseline and change induced by iTBS. Plots of the relationship between TMS-related theta (A), beta (B) and gamma (C) power at baseline and changes after iTBS. Change scores were calculated by subtracting baseline (pre-iTBS) power from post-iTBS power values. In each frequency band, baseline power significantly predicted change scores. In the gamma band alone, the interaction effect turned significant as well.

### 3.4 Microstates

Lastly, to evaluate effects of the stimulation protocols on resting-state EEG, descriptive MS parameters were analyzed (Table 2). Descriptive parameters of the post-iTBS measurement are reported as changes relative to the baseline by subtracting the baseline from post-iTBS values.

**Table 2.** Descriptive statistics of Microstate analysis.

	Microstate A	Microstate B	Microstate C	Microstate D
Occurrence (per sec)				
Pre active	3.644 (0.514)	3.768 (0.507)	4.191 (0.498)	4.110 (0.552)
∆ Active	+ 0.001 (0.279)	− 0.213 (0.397)	− 0.111 (0.401)	− 0.130 (0.269)
Pre sham	3.662 (0.554)	3.748 (0.392)	4.124 (0.344)	4.143 (0.544)
∆ Sham	− 0.064 (0.325)	− 0.221 (0.460)	− 0.132 (0.306)	− 0.136 (0.401)
∆ Overall	− 0.031 (0.305)	− 0.217 (0.430)**	− 0.122 (0.357)*	− 0.133 (0.341)*
Duration (ms)				
Pre active	60.166 (5.089)	61.223 (7.130)	70.952 (12.122)	62.366 (7.040)
∆ Active	+ 2.563 (7.302)	+ 1.031 (5.607)	+ 2.410 (7.873)	+ 1.608 (6.096)
Pre sham	61.042 (5.940)	61.395 (7.052)	69.861 (9.839)	62.985 (6.010)
∆ Sham	+ 2.380 (5.219)	+1.588 (4.484)	+ 3.213 (8.726)	+ 2.068 (5.751)
∆ Overall	+ 2.472 (4.799)**	+ 1.310 (5.249)	+ 2.812 (8.320)	+ 1.838 (5.930)
Coverage (%)				
Pre active	21.783 (2.616)	23.019 (3.649)	29.653 (5.639)	25.545 (3.776)
∆ Active	+ 0.884 (2.087)	− 0.920 (3.694)	+ 0.288 (4.649)	− 0.252 (3.211)
Pre sham	22.270 (3.466)	22.926 (2.957)	28.746 (4.337)	26.058 (3.853)
∆ Sham	+ 0.408 (2.010)	− 0.777 (3.489)	+ 0.493 (4.023)	− 0.123 (2.823)
∆ Overall	+ 0.646 (2.062)*	−0.849 (3.593)	+ 0.390 (4.348)	−0.187 (3.024)

*Note.*Parameters of occurrence, duration and coverage are listed per MS A, B, C and D. Post-iTBS parameters are reported as the difference when subtracting the baseline values from post-iTBS values (∆), standard deviations are reported in brackets. *p≤ 0.05, **p≤ 0.01.

The RM two-way ANOVA with the factors time (baseline vs. post) and condition (active vs. sham) resulted in no significant interaction effects but multiple significant main effects of time.

For MS A, both duration (*F*(1,24) = 9.640,*p*= 0.0047) and coverage (*F*(1,24) = 4.735,*p*= 0.039) increased significantly post-iTBS. MS B (*F*(1,24) = 7.936,*p*= 0.009), C (*F*(1,24) = 5.586,*p*= 0.026) and D (*F*(1,24) = 5.548,*p*= 0.027) all occurred significantly less post-iTBS.

## 4 Discussion

In the current study, we evaluated effects of a concurrent phase-locked tACS-iTBS stimulation protocol on the excitability of the DLPFC by comparing TMS-evoked activity in the EEG and resting-state microstate analysis. A general effect of both the active and sham tACS-iTBS protocols was observable on most markers investigated. Additional effects of the active tACS-iTBS stimulation protocol were observed in measures of GMFP and predictive power of baseline activity on excitability changes in the gamma frequency band.

While we found a significant reduction in sub-threshold GMFP in both post measurements between 200–260 ms and 320–380 ms, the tACS-iTBS condition additionally resulted in a significant suppression between 120–140 ms, which was not the case after the sham tACS stimulation. This suppression is in a time frame of the N100 component, which has been suggested to represent an auditory and somatosensory response to the TMS pulses, finding similar responses to sham and real TMS stimulation (Conde et al., 2019) and auditory stimulation and unmasked TMS stimulation (Rocchi et al., 2021). Other studies, however, found that this N100 component with a topographical distribution around the vertex showed a significantly higher amplitude after stimulation compared to sham (Du at al., 2017), although significantly reduced when compared to proper noise-masking (Poorganji et al., 2023). While the N100 component in our results could still contain auditory and somatosensory artifacts, specific changes after the tACS-iTBS was unlikely explained by sensory-evoked N100 component, but rather explained by the tACS-iTBS protocol. Additionally, this interaction effect was absent after supra-threshold TMS. Possible explanations for this absence are that the high-intensity TMS saturated the subtle differences modulated by the tACS-iTBS protocol, or that the artifacts induced by the TMS overpowered the non-auditory and non-somatosensory neuronal responses. Our channel-wise analysis resulted in a decrease in this N100 around the vertex between 120–160 ms in the post-iTBS measurement, representing a general effect of iTBS on this N100. Contrary to the GMFP results, no significant interaction was observed in the channel-wise analysis. These results suggest that while our tACS-iTBS stimulation protocol didn’t have an additional influence on localized cortical excitability compared to iTBS alone, although global changes could be observed.

Interestingly, a study examining effects of iTBS in a sample of patients with treatment-resistant depression found a similar suppression in GMFP amplitude around 100 ms, but only in patients classified as responders of iTBS treatment for MDD (Strafella et al., 2023). This could indicate that our tACS-iTBS stimulation protocol succeeded in creating a bias to LTP induction and possibly improve iTBS treatment effect for MDD.

The significant main effects of time in all evaluated frequency bands support a general effect of iTBS on TMS-related oscillatory power. The concurrent tACS-iTBS stimulation seemed to only have a small effect on oscillatory power, with the interaction between time and condition only turning significant for oscillations in the beta frequency. Overall, we found a general trend for TMS-related power in the gamma, beta and theta frequency bands to be suppressed after both tACS-iTBS and sham-tACS-iTBS, whileBriley et al. (2024)found a significant increase in endogenous frontal theta power during a n-back working memory task after application of the same tACS-iTBS stimulation protocol. Regarding iTBS-induced changes in TMS-related power, two studies (Chung et al., 2017,2018b) have found a TMS-related theta power increase, while one other study found a decrease (Desforges et al., 2022). The high variability between participants in TMS-related oscillatory power at baseline could provide an answer to why (1) studies reported contradictory results and (2) iTBS has a facilitatory effect on excitability in some cases but not in others.

Results of the regression indicate that baseline values in a specific frequency band significantly affect the changes of TMS-related oscillations induced by iTBS application. The relationship seems to point in the direction that iTBS will result in an increase in oscillatory power in subjects with lower initial TMS-related oscillatory power. With baseline power values increasing, oscillatory power decreases post-iTBS. Baseline oscillatory power interacted significantly with the stimulation protocols only in the gamma frequency. This suggests that the relationship between baseline and post-iTBS values got reinforced by our concurrent phase-locked tACS-iTBS stimulation protocol. A study pooling TMS-EEG data across multiple studies (Corp et al., 2020) found a similar influence of iTBS to the primary motor cortex (M1) on motor-evoked potentials (MEPs). Smaller baseline MEPs related to larger post-iTBS MEP amplitudes. The same result was found in another study (Leodori et al., 2021), with the addition that iTBS evoked beta power significantly predicted MEP facilitation as well. The authors explain their results with the concept of homeostatic metaplasticity, which assumes that a lower baseline synaptic activity is associated with a higher probability of inducing LTP while higher baseline synaptic activity is associated with a lower probability of inducing LTP. This refers to regulation of plasticity in the brain to avoid overshooting LTP or LTD and keeping networks in a physiological range (Abraham, 2008;Müller-Dahlhaus & Ziemann, 2014). These results indicate that there is a physiological limit to how much cortical activity can be influenced by non-invasive brain stimulation methods such as iTBS.

In our analysis of resting-state data, we observed significant changes in all MS evaluated as a result of the iTBS stimulation, but no interaction effects. MS A exhibited a significant increase in both duration and coverage of the MS B, C and D on the other hand all occurred significantly less post-iTBS. To date, not many studies have evaluated the effects of iTBS to the lDLPFC on microstate parameters.Gold et al. (2022)applied high-frequency rTMS to the lDLPFC in a patient sample with major depressive disorder and found increased coverage and occurrence of what they labeled MS 2 as well as decreased coverage and occurrence of MS 3. These changes only happened in patients responding to the treatment and correlated with clinical response. The authors labeled their MS 2 as MS C and MS 3 as MS D. Visually, their MS 2 is comparable to the MS A extracted from our data and their MS 3 is comparable to our MS D. The results are not directly comparable becauseGold et al. (2022)evaluated long-term effects of rTMS on a clinical sample whereas in the present study, short-term effects of iTBS were evaluated in a healthy sample. The absence of any effects on MS in the non-responders of the Gold and colleagues study suggests that our participants seemed to respond to iTBS and changes observed in MS A, B, C, and D might be representative of short-term iTBS-induced MS changes in a healthy population.Custo et al. (2017)conducted source localization analysis on microstates. They found maximal activation in the left Heschl’s gyrus, left Wernicke area, left insula, and left lingual gyrus for MS A, Cuneus, right Insula, right claustrum, and right frontal eye field for MS B, Precuneus, posterior cingulate cortex and left angular gyrus for MS C and right inferior parietal lobe, right mid and superior frontal gyrus as well as right insula for MS D. In the case of our study, the observed increase MS A duration and coverage might therefore represent a shift in bias for networks closer to the area of stimulation as they seem to include mostly left-hemispheric temporal regions. MS B, C, and D on the other hand are thus reduced accordingly, as they are located further away of the stimulated DLPFC, either in the right hemisphere or in a more posterior area.

There are several limitations in the current study. We did not employ a sham-sham condition, where both tACS and iTBS were not truly applied. This makes it impossible to rule out whether some of the effects were simply based on time passing between the baseline and post-iTBS measurements. The goal of the present study however was to determine additional effects of pairing iTBS in a phase-locked manner to a concurrent tACS current. Previous studies have examined the effect of iTBS alone, comparing it with a pure sham condition (Chung et al., 2018a,2018b,^2019^). Additionally, we found a significant interaction effect in our GMFP analysis, which can’t be explained by effects of arousal or similar.

Additionally, the resting-state, sub- and supra-threshold measurements were always applied in the same order before and after the stimulation protocols. This means that resting-state was measured around 5 minutes after iTBS, sub-threshold 10 minutes, and supra-threshold 15 minutes after iTBS. Accordingly, effects of time elapsed after stimulation can’t be ruled out to have had a significant effect on the corresponding measurements, which had a significant effect on results in other cases (Briley et al., 2024). Additionally, we only tested one phase of the tACS current where iTBS pulses were applied. In particular, iTBS locked to the troughs of tACS could modulate cortical excitability in a different manner.

Despite the gold standard of assessing the motor threshold being the use of electromyography (EMG), we utilized visual observation of a finger twitch to determine the aMT. While this is a less accurate assessment of aMT, the M1 aMT itself is only an approximation for DLPFC stimulation.

Moreover, using the 10–20 EEG system to target the left DLPFC is only a very approximate method. The precision of a TMS target would be improved by using a neuronavigational device with individual structural MRIs of participants. In the current study, however, the constraints of the experimental setting of applying HD-tACS through our EEG electrodes made individual targeting not possible. In future studies, this could be overridden by excluding EEG measurements, thus allowing to place the HD-tACS montage also with a neuronavigational device.

Regarding the preprocessing pipeline, our epoch length of 1,000 ms is suboptimal for achieving a high-quality time-frequency decomposition in the lower frequency bands. The use of Morlet wavelet convolution inherently introduces temporal smearing, an effect which is particularly pronounced at lower frequencies where wavelets have longer temporal durations. With our relatively short epoch length from -500 to 500 ms around the TMS pulse, the wavelet kernels for these lower frequencies extend across both the pre- and post-TMS periods. This overlap could lead to an overestimation of baseline activity and underestimation of TMS-related responses. Additionally, concatenating trials prior to conducting time-frequency decomposition amplifies this issue, as post-TMS activity from one trial can blend into the baseline activity of the following trial, and vice-versa, further blurring the distinction between baseline and TMS-related activity. However, restricting the epoch length to this window improved the removal of eye artifacts during preprocessing, thereby enhancing overall data quality. Furthermore, using fewer cycles at lower frequencies allowed for better temporal localization, reducing temporal smearing and improving the time-resolution of low-frequency activity.

## 5 Conclusion

Our results demonstrated that our concurrent phase-locked tACS-iTBS stimulation protocol has an additional effect compared to iTBS to modulate overall power across the whole scalp as seen in a decrease in N100 amplitude of the GMFP. The absence of channel-wise effects suggest a more global effect on LTP. Additionally, baseline excitability might play a role for contradictory results how iTBS affects TMS-EEG measures like oscillatory power. This could indicate that if baseline excitability is suppressed in a clinical population, the stimulation protocol might produce more explicit results. Future studies should thus investigate the effects of this concurrent, phase-locked tACS-iTBS protocol in a clinical population over multiple sessions to assess effects on individuals with aberrant cortical excitability and evaluate long-term changes to plasticity.

## Supplementary Material

Supplementary Material

## Data Availability

The data and code used in the present study are available upon request from the corresponding author given the presence of a formal data sharing agreement.

## References

[b200] Abraham , W. C. ( 2008 ). Metaplasticity: Tuning synapses and networks for plasticity . Nature Reviews Neuroscience , 9 ( 5 ), 387 – 387 . 10.1038/nrn2356 18401345

[b1] Atluri , S. , Frehlich , M. , Mei , Y. , Garcia Dominguez , L., Rogasch , N. C. , Wong , W. , & Farzan , F. ( 2016 ). TMSEEG: A MATLAB-based graphical user interface for processing electrophysiological signals during transcranial magnetic stimulation . Frontiers in Neural Circuits , 10 , 78 . 10.3389/fncir.2016.00078 27774054 PMC5054290

[b2] Baur , D. , Galevska , D. , Hussain , S. , Cohen , L. G. , Ziemann , U. , & Zrenner , C. ( 2020 ). Induction of LTD-like corticospinal plasticity by low-frequency rTMS depends on pre-stimulus phase of sensorimotor μ-rhythm . Brain Stimulation , 13 ( 6 ), 1580 – 1587 . 10.1016/j.brs.2020.09.005 32949780 PMC7710977

[b3] Briley , P. M. , Boutry , C. , Webster , L. , Veniero , D. , Harvey-Seutcheu , C. , Jung , J. , & Morriss , R. ( 2024 ). Intermittent theta burst stimulation with synchronised transcranial alternating current stimulation leads to enhanced frontal theta oscillations and a positive shift in emotional bias . Imaging Neuroscience , 2 , 1 – 14 . 10.1162/imag_a_00073 PMC12224432 40800403

[b4] Chung , S. W. , Lewis , B. P. , Rogasch , N. C. , Saeki , T. , Thomson , R. H. , Hoy , K. E. , & Fitzgerald , P. B. ( 2017 ). Demonstration of short-term plasticity in the dorsolateral prefrontal cortex with theta burst stimulation: A TMS-EEG study . Clinical Neurophysiology , 128 ( 7 ), 1117 – 1126 . 10.1016/j.clinph.2017.04.005 28511124

[b5] Chung , S. W. , Rogasch , N. C. , Hoy , K. E. , & Fitzgerald , P. B. ( 2018a ). The effect of single and repeated prefrontal intermittent theta burst stimulation on cortical reactivity and working memory . Brain Stimulation , 11 ( 3 ), 566 – 574 . 10.1016/j.brs.2018.01.002 29352668

[b6] Chung , S. W. , Rogasch , N. C. , Hoy , K. E. , Sullivan , C. M. , Cash , R. F. , & Fitzgerald , P. B. ( 2018b ). Impact of different intensities of intermittent theta burst stimulation on the cortical properties during TMS‐EEG and working memory performance . Human Brain Mapping , 39 ( 2 ), 783 – 802 . 10.1002/hbm.23882 29124791 PMC6866298

[b7] Chung , S. W. , Sullivan , C. M. , Rogasch , N. C. , Hoy , K. E. , Bailey , N. W. , Cash , R. F. , & Fitzgerald , P. B. ( 2019 ). The effects of individualised intermittent theta burst stimulation in the prefrontal cortex: A TMS‐EEG study . Human Brain Mapping , 40 ( 2 ), 608 – 627 . 10.1002/hbm.24398 30251765 PMC6865598

[b8] Conde , V. , Tomasevic , L. , Akopian , I. , Stanek , K. , Saturnino , G. B. , Thielscher , A. , & Siebner , H. R. ( 2019 ). The non-transcranial TMS-evoked potential is an inherent source of ambiguity in TMS-EEG studies . NeuroImage , 185 , 300 – 312 . 10.1016/j.neuroimage.2018.10.052 30347282

[b9] Corp , D. T. , Bereznicki , H. G. , Clark , G. M. , Youssef , G. J. , Fried , P. J. , Jannati , A. , & Enticott , P. G. ( 2020 ). Large-scale analysis of interindividual variability in theta-burst stimulation data: Results from the ‘Big TMS Data Collaboration’ . Brain Stimulation , 13 ( 5 ), 1476 – 1488 . 10.1016/j.brs.2020.07.018 32758665 PMC7494610

[b10] Croce , P. , Zappasodi , F. , & Capotosto , P. ( 2018 ). Offline stimulation of human parietal cortex differently affects resting EEG microstates . Scientific Reports , 8 ( 1 ), 1287 . 10.1038/s41598-018-19698-z 29352255 PMC5775423

[b11] Cruciani , A. , Mancuso , M. , Sveva , V. , Maccarrone , D. , Todisco , A. , Motolese , F. , & Capone , F. ( 2023 ). Using TMS-EEG to assess the effects of neuromodulation techniques: A narrative review . Frontiers in Human Neuroscience , 17 , 1247104 . 10.3389/fnhum.2023.1247104 37645690 PMC10461063

[b12] Custo , A. , Van De Ville , D., Wells , W. M. , Tomescu , M. I. , Brunet , D. , & Michel , C. M. ( 2017 ). Electroencephalographic resting-state networks: Source localization of microstates . Brain Connectivity , 7 ( 10 ), 671 – 682 . 10.1089/brain.2016.0476 28938855 PMC5736178

[b13] Delorme , A. , & Makeig , S. ( 2004 ). EEGLAB: An open source toolbox for analysis of single-trial EEG dynamics including independent component analysis . Journal of Neuroscience Methods , 134 ( 1 ), 9 – 21 . 10.1016/j.jneumeth.2003.10.009 15102499

[b14] Desforges , M. , Hadas , I. , Mihov , B. , Morin , Y. , Braün , M. R. , Lioumis , P. , & Tremblay , S. ( 2022 ). Dose-response of intermittent theta burst stimulation of the prefrontal cortex: A TMS-EEG study . Clinical Neurophysiology , 136 , 158 – 172 . 10.1016/j.clinph.2021.12.018 35183861

[b15] Ding , X. , Li , X. , Xu , M. , He , Z. , & Jiang , H. ( 2023 ). The effect of repetitive transcranial magnetic stimulation on electroencephalography microstates of patients with heroin-addiction . Psychiatry Research: Neuroimaging , 329 , 111594 . 10.1016/j.pscychresns.2023.111594 36724624

[b16] Ding , Z. , Wang , Y. , Li , J. , & Li , X. ( 2022 ). Closed-loop TMS-EEG reactivity with occipital alpha-phase synchronized . Journal of Neural Engineering , 19 ( 5 ), 056027 . 10.1088/1741-2552/ac9432 36137522

[b17] Dissanayaka , T. D. , Zoghi , M. , Farrell , M. , Egan , G. F. , & Jaberzadeh , S. ( 2018 ). Sham transcranial electrical stimulation and its effects on corticospinal excitability: A systematic review and meta-analysis . Reviews in the Neurosciences , 29 ( 2 ), 223 – 232 . 10.1515/revneuro-2017-0026 28889119

[b18] Drabant , E. M. , McRae , K. , Manuck , S. B. , Hariri , A. R. , & Gross , J. J. ( 2009 ). Individual differences in typical reappraisal use predict Amygdala and prefrontal responses . Biological Psychiatry , 65 ( 5 ), 367 – 373 . 10.1016/j.biopsych.2008.09.007 18930182 PMC2855682

[b19] Du , X. , Choa , F. S. , Summerfelt , A. , Rowland , L. M. , Chiappelli , J. , Kochunov , P. , & Hong , L. E. ( 2017 ). N100 as a generic cortical electrophysiological marker based on decomposition of TMS-evoked potentials across five anatomic locations . Experimental Brain Research , 235 , 69 – 81 . 10.1007/s00221-016-4773-7 27628235 PMC5269602

[b20] Faller , J. , Doose , J. , Sun , X. , Mclntosh , J. R. , Saber , G. T. , Lin , Y. , & Sajda , P. ( 2022 ). Daily prefrontal closed-loop repetitive transcranial magnetic stimulation (rTMS) produces progressive EEG quasi-alpha phase entrainment in depressed adults . Brain Stimulation , 15 ( 2 ), 458 – 471 . 10.1016/j.brs.2022.02.008 35231608 PMC8979612

[b21] Fehér , K. D. , Nakataki , M. , & Morishima , Y. ( 2017 ). Phase-dependent modulation of signal transmission in cortical networks through tACS-induced neural oscillations . Frontiers in Human Neuroscience , 11 , 471 . 10.3389/fnhum.2017.00471 29021749 PMC5624081

[b22] Gbadeyan , O. , McMahon , K. , Steinhauser , M. , & Meinzer , M. ( 2016 ). Stimulation of dorsolateral prefrontal cortex enhances adaptive cognitive control: A high-definition transcranial direct current stimulation study . Journal of Neuroscience , 36 ( 50 ), 12530 – 12536 . 10.1523/JNEUROSCI.2450-16.2016 27974612 PMC6705663

[b23] Gold , M. C. , Yuan , S. , Tirrell , E. , Kronenberg , E. F. , Kang , J. W. D. , Hindley , L. , & Carpenter , L. L. ( 2022 ). Large-scale EEG neural network changes in response to therapeutic TMS . Brain Stimulation , 15 ( 2 ), 316 – 325 . 10.1016/j.brs.2022.01.007 35051642 PMC8957581

[b24] Goldin , P. R. , McRae , K. , Ramel , W. , & Gross , J. J. ( 2008 ). The neural bases of emotion regulation: Reappraisal and suppression of negative emotion . Biological Psychiatry , 63 ( 6 ), 577 – 586 . 10.1016/j.biopsych.2007.05.031 17888411 PMC2483789

[b25] Guerra , A. , López-Alonso , V. , Cheeran , B. , & Suppa , A. ( 2020 ). Variability in non-invasive brain stimulation studies: Reasons and results . Neuroscience Letters , 719 , 133330 . 10.1016/j.neulet.2017.12.058 29294333

[b26] Guerra , A. , Suppa , A. , Bologna , M. , D’Onofrio , V. , Bianchini , E. , Brown , P. , & Berardelli , A. ( 2018 ). Boosting the LTP-like plasticity effect of intermittent theta-burst stimulation using gamma transcranial alternating current stimulation . Brain Stimulation , 11 ( 4 ), 734 – 742 . 10.1016/j.brs.2018.03.015 29615367 PMC6022811

[b27] Guo , Y. , Zhao , X. , Liu , X. , Liu , J. , Li , Y. , Yue , L. , & Yuan , K. ( 2023 ). Electroencephalography microstates as novel functional biomarkers for insomnia disorder . General Psychiatry , 36 ( 6 ), e101171 . 10.1136/gpsych-2023-101171 38143715 PMC10749048

[b28] Habermann , M. , Weusmann , D. , Stein , M. , & Koenig , T. ( 2018 ). A student’s guide to randomization statistics for multichannel event-related potentials using Ragu . Frontiers in Neuroscience , 12 , 359374. 10.3389/fnins.2018.00355 PMC6020783 29973861

[b29] Hamada , M. , Murase , N. , Hasan , A. , Balaratnam , M. , & Rothwell , J. C. ( 2013 ). The role of interneuron networks in driving human motor cortical plasticity . Cerebral Cortex , 23 ( 7 ), 1593 – 1605 . 10.1093/cercor/bhs147 22661405

[b30] Helfrich , R. F. , Schneider , T. R. , Rach , S. , Trautmann-Lengsfeld , S. A. , Engel , A. K. , & Herrmann , C. S. ( 2014 ). Entrainment of brain oscillations by transcranial alternating current stimulation . Current Biology , 24 ( 3 ), 333 – 339 . 10.1016/j.cub.2013.12.041 24461998

[b31] Huang , Y. Z. , Edwards , M. J. , Rounis , E. , Bhatia , K. P. , & Rothwell , J. C. ( 2005 ). Theta burst stimulation of the human motor cortex . Neuron , 45 ( 2 ), 201 – 206 . 10.1016/j.neuron.2004.12.033 15664172

[b32] Ilmoniemi , R. J. , & Kičić , D. ( 2010 ). Methodology for combined TMS and EEG . Brain Topography , 22 , 233 – 248 . 10.1007/s10548-009-0123-4 20012350 PMC2800178

[b33] Kanai , R. , Chaieb , L. , Antal , A. , Walsh , V. , & Paulus , W. ( 2008 ). Frequency-dependent electrical stimulation of the visual cortex . Current Biology , 18 ( 23 ), 1839 – 1843 . 10.1016/j.cub.2008.10.027 19026538

[b34] Klomjai , W. , Katz , R. , & Lackmy-Vallée , A. ( 2015 ). Basic principles of transcranial magnetic stimulation (TMS) and repetitive TMS (rTMS) . Annals of Physical and Rehabilitation Medicine , 58 ( 4 ), 208 – 213 . 10.1016/j.rehab.2015.05.005 26319963

[b35] Koenig , T. , & Melie-Garcia , L. ( 2010 ). A method to determine the presence of averaged event-related fields using randomization tests . Brain Topography , 23 , 233 – 242 . 10.1007/s10548-010-0142-1 20376546

[b36] Lefaucheur , J. P. , Aleman , A. , Baeken , C. , Benninger , D. H. , Brunelin , J. , Di Lazzaro , V., & Ziemann , U. ( 2020 ). Evidence-based guidelines on the therapeutic use of repetitive transcranial magnetic stimulation (rTMS): An update (2014–2018) . Clinical Neurophysiology , 131 ( 2 ), 474 – 528 . 10.1016/j.clinph.2019.11.002 31901449

[b37] Lehmann , D. , Ozaki , H. , & Pál , I. ( 1987 ). EEG alpha map series: Brain micro-states by space-oriented adaptive segmentation . Electroencephalography and Clinical Neurophysiology , 67 ( 3 ), 271 – 288 . 10.1016/0013-4694(87)90025-3 2441961

[b38] Lehmann , D. , & Skrandies , W. ( 1980 ). Reference-free identification of components of checkerboard-evoked multichannel potential fields . Electroencephalography and Clinical Neurophysiology , 48 ( 6 ), 609 – 621 . 10.1016/0013-4694(80)90419-8 6155251

[b39] Leodori , G. , Fabbrini , A. , De Bartolo , M. I. , Costanzo , M. , Asci , F. , Palma , V. , & Berardelli , A. ( 2021 ). Cortical mechanisms underlying variability in intermittent theta-burst stimulation-induced plasticity: A TMS-EEG study . Clinical Neurophysiology , 132 ( 10 ), 2519 – 2531 . 10.1016/j.clinph.2021.06.021 34454281

[b40] Luo , X. , Che , X. , & Li , H. ( 2023 ). Concurrent TMS-EEG and EEG reveal neuroplastic and oscillatory changes associated with self-compassion and negative emotions . International Journal of Clinical and Health Psychology , 23 ( 1 ), 100343 . 10.1016/j.ijchp.2022.100343 36299492 PMC9577271

[b41] MacDonald , A. W. , Cohen , J. D. , Stenger , V. A. , & Carter , C. S. ( 2000 ). Dissociating the role of the dorsolateral prefrontal and anterior cingulate cortex in cognitive control . Science , 288 ( 5472 ), 1835 – 1838 . 10.1126/science.288.5472.1835 10846167

[b42] Maiella , M. , Casula , E. P. , Borghi , I. , Assogna , M. , D’Acunto , A. , Pezzopane , V. , & Koch , G. ( 2022 ). Simultaneous transcranial electrical and magnetic stimulation boost gamma oscillations in the dorsolateral prefrontal cortex . Scientific Reports , 12 ( 1 ), 19391 . 10.1038/s41598-022-23040-z 36371451 PMC9653481

[b43] Michel , C. M. , & Koenig , T. ( 2018 ). EEG microstates as a tool for studying the temporal dynamics of whole-brain neuronal networks: A review . NeuroImage , 180 , 577 – 593 . 10.1016/j.neuroimage.2017.11.062 29196270

[b201] Müller-Dahlhaus , F. , & Ziemann , U. ( 2015 ). Metaplasticity in human cortex . The Neuroscientist , 21 ( 2 ), 185 – 202 . 10.1177/1073858414526645 24620008

[b44] Nagabhushan Kalburgi, S. , Kleinert , T. , Aryan , D. , Nash , K. , Schiller , B. , & Koenig , T. ( 2023 ). MICROSTATELAB: The EEGLAB Toolbox for resting-state Microstate Analysis . Brain Topography , 37 , 621 – 645 . 10.1007/s10548-023-01003-5 37697212 PMC11199309

[b45] Nigbur , R. , Cohen , M. X. , Ridderinkhof , K. R. , & Stürmer , B. ( 2012 ). Theta dynamics reveal domain-specific control over stimulus and response conflict . Journal of Cognitive Neuroscience , 24 ( 5 ), 1264 – 1274 . 10.1162/jocn_a_00128 21861681

[b46] Pan , Z. , Xiong , D. , Xiao , H. , Li , J. , Huang , Y. , Zhou , J. , & Wu , K. ( 2021 ). The effects of repetitive transcranial magnetic stimulation in patients with chronic schizophrenia: Insights from EEG microstates . Psychiatry Research , 299 , 113866 . 10.1016/j.psychres.2021.113866 33735740

[b47] Poorganji , M. , Zomorrodi , R. , Hawco , C. , Hill , A. T. , Hadas , I. , Zrenner , C. , & Daskalakis , Z. J. ( 2023 ). Isolating sensory artifacts in the suprathreshold TMS-EEG signal over DLPFC . Scientific Reports , 13 ( 1 ), 6796 . 10.1038/s41598-023-29920-2 37100795 PMC10130812

[b48] Qiu , S. , Wang , S. , Peng , W. , Yi , W. , Zhang , C. , Zhang , J. , & He , H. ( 2022 ). Continuous theta-burst stimulation modulates resting-state EEG microstates in healthy subjects . Cognitive Neurodynamics , 16 , 621 – 631 . 10.1007/s11571-021-09726-6 35603056 PMC9120322

[b49] Ridderinkhof , K. R. , van den Wildenberg , W. P. , Segalowitz , S. J. , & Carter , C. S. ( 2004 ). Neurocognitive mechanisms of cognitive control: The role of prefrontal cortex in action selection, response inhibition, performance monitoring, and reward-based learning . Brain and Cognition , 56 ( 2 ), 129 – 140 . 10.1016/j.bandc.2004.09.016 15518930

[b50] Rocchi , L. , Di Santo , A., Brown , K. , Ibánez , J. , Casula , E. , Rawji , V. , & Rothwell , J. ( 2021 ). Disentangling EEG responses to TMS due to cortical and peripheral activations . Brain Stimulation , 14 ( 1 ), 4 – 18 . 10.1016/j.brs.2020.10.011 33127580

[b51] Rosanova , M. , Casali , A. , Bellina , V. , Resta , F. , Mariotti , M. , & Massimini , M. ( 2009 ). Natural frequencies of human corticothalamic circuits . Journal of Neuroscience , 29 ( 24 ), 7679 – 7685 . 10.1523/JNEUROSCI.0445-09.2009 19535579 PMC6665626

[b52] Rossi , S. , Hallett , M. , Rossini , P. M. , & Pascual-Leone , A. ( 2011 ). Screening questionnaire before TMS: An update . Clinical Neurophysiology , 122 ( 8 ), 1686 . 10.1016/j.clinph.2010.12.037 21227747

[b53] Rossi , S. , Hallett , M. , Rossini , P. M. , Pascual-Leone , A. , & Safety of TMS Consensus Group . ( 2009 ). Safety, ethical considerations, and application guidelines for the use of transcranial magnetic stimulation in clinical practice and research . Clinical Neurophysiology , 120 ( 12 ), 2008 – 2039 . 10.1016/j.clinph.2009.08.016 19833552 PMC3260536

[b54] Strafella , R. , Momi , D. , Zomorrodi , R. , Lissemore , J. , Noda , Y. , Chen , R. , & Voineskos , D. ( 2023 ). Identifying neurophysiological markers of intermittent theta burst stimulation in treatment-resistant depression using transcranial magnetic stimulation–electroencephalography . Biological Psychiatry , 94 ( 6 ), 454 – 465 . 10.1016/j.biopsych.2023.04.011 37084864

[b55] Sverak , T. , Albrechtova , L. , Lamos , M. , Rektorova , I. , & Ustohal , L. ( 2018 ). Intensive repetitive transcranial magnetic stimulation changes EEG microstates in schizophrenia: A pilot study . Schizophrenia Research , 193 , 451 – 452 . 10.1016/j.schres.2017.06.044 28673751

[b56] Villamar , M. F. , Volz , M. S. , Bikson , M. , Datta , A. , DaSilva , A. F. , & Fregni , F. ( 2013 ). Technique and considerations in the use of 4 x 1 ring high-definition transcranial direct current stimulation (HD-tDCS) . Journal of Visualized Experiments , 77 , e50309 . 10.3791/50309 PMC3735368 23893039

[b57] Vöröslakos , M. , Takeuchi , Y. , Brinyiczki , K. , Zombori , T. , Oliva , A. , Fernández-Ruiz , A. , & Berényi , A. ( 2018 ). Direct effects of transcranial electric stimulation on brain circuits in rats and humans . Nature Communications , 9 ( 1 ), 483 . 10.1038/s41467-018-02928-3 PMC5797140 29396478

[b58] Zaehle , T. , Rach , S. , & Herrmann , C. S. ( 2010 ). Transcranial alternating current stimulation enhances individual alpha activity in human EEG . PLoS One , 5 ( 11 ), e13766 . 10.1371/journal.pone.0013766 21072168 PMC2967471

[b59] Zhao , L. , Zhou , D. , Hu , J. , He , X. , Peng , X. , Ma , L. , & Kuang , L. ( 2023 ). Changes in microstates of first-episode untreated nonsuicidal self-injury adolescents exposed to negative emotional stimuli and after receiving rTMS intervention . Frontiers in Psychiatry , 14 , 1151114 . 10.3389/fpsyt.2023.1151114 37181884 PMC10172670

