# Supplementary material for: Enhancing prefrontal modulation by phase-locking intermittent theta burst stimulation to a concurrent transcranial alternating current stimulation
Source: Imaging Neurosci (Camb). 2025 Jan 3;3:imag_a_00415. doi: 10.1162/imag_a_00415 (PMC7617709; doi:10.1162/imag_a_00415)
Supplement: Supplementary Material [file imag_a_00415-supp.pdf]

## Supplementary Materials

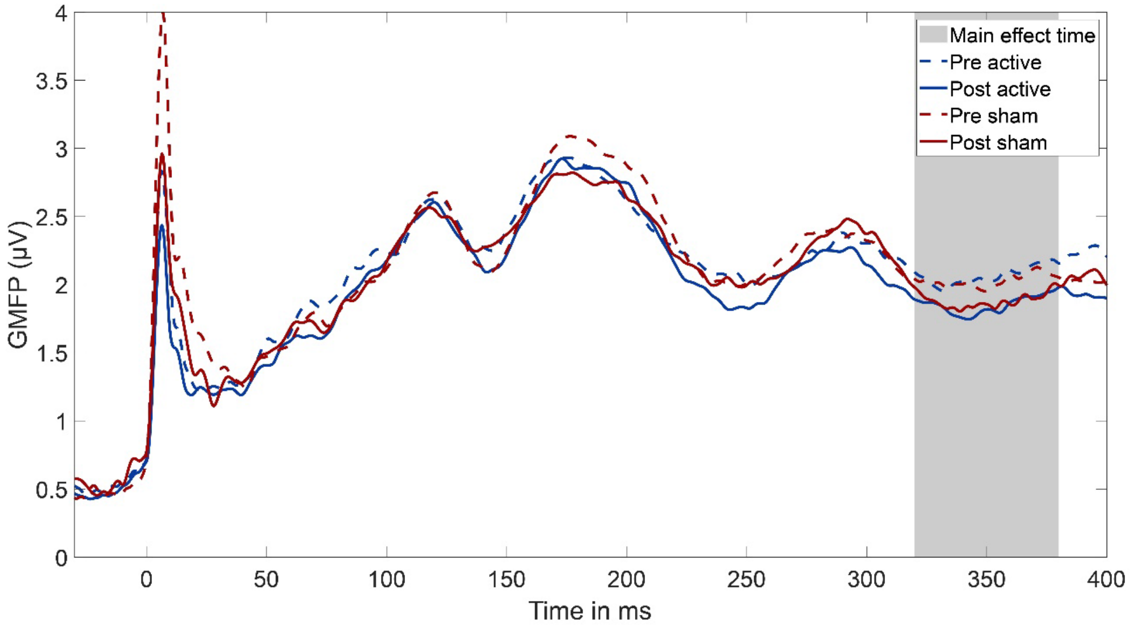


Supplementary Figure 1. Global Mean Field Power after supra-threshold TMS.

Global Mean Field Power of TMS-evoked potentials across the scalp. Baseline (pre-) and post-iTBS are depicted separately for the active and sham conditions. Time periods marked in light grey depict a significant main effect of the active and sham stimulation conditions. The RM two-way ANOVA yielded no significant interaction effects.

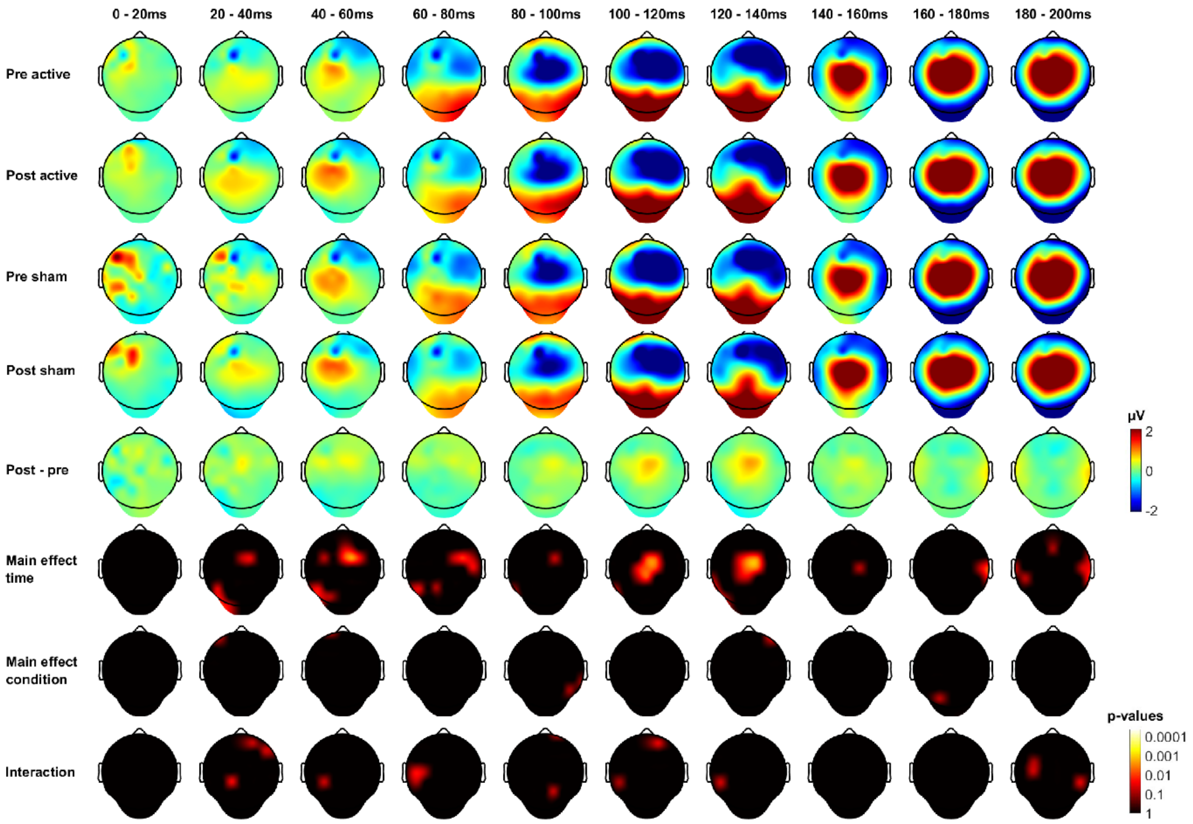


Supplementary Figure 2. Results of electrode-wise analysis for the supra-threshold TMS-evoked potentials. Separate depiction of all conditions and results of the electrode-wise RM two-way ANOVA.
